# Metabolic–Epigenetic Crosstalk in Takayasu Arteritis: The ANK2–MAVS–IL-8 Axis as a Novel Therapeutic Paradigm

**DOI:** 10.3390/ijms27073249

**Published:** 2026-04-03

**Authors:** Tianjian Xie, Leyu Zhang, Shurong Li, Benmo Xu, Xinyu Zhang, Yajun Wang, Zixiang Shang, Hongxuan Xi, Han Shi, Xin Ni, Ping Li, Hengxi Li

**Affiliations:** 1 Department of Anatomy and Histology/Embryology, Faculty of Basic Medical Sciences, Kunming Medical University, Kunming 650500, China; 13649669084@163.com (T.X.); 18288932389@163.com (L.Z.); 18087340634@163.com (X.Z.); 19956113973@163.com (Y.W.); ascea_112@163.com (X.N.); 2 The First Clinical Medical College, Kunming Medical University, Kunming 650032, China; 18887265770@163.com (S.L.); 18314459781@163.com (Z.S.); 15887995418@163.com (H.X.); 14787119305@163.com (H.S.); 3 School of Clinical Oncology, Kunming Medical University, Kunming 650118, China; 2022542053@kmmu.edu.cn

**Keywords:** Takayasu arteritis, metabolic reprogramming, epigenetic regulation, mitochondrial stress, ANK2–MAVS–IL-8 axis, vascular inflammation, biomarkers

## Abstract

Takayasu arteritis (TAK) is a refractory chronic vasculitis of the aorta and its major branches, characterized by unsatisfactory treatment responses and high relapse rates. This review synthesizes current evidence to propose and elaborate a novel pathogenic paradigm: a self-reinforcing “metabolism–epigenetics–inflammation” feedback loop that sustains chronic vascular inflammation in TAK. We detail how immunometabolic reprogramming in immune and vascular wall cells not only meets bioenergetic demands but also generates metabolites (e.g., acetyl-CoA, lactate) that serve as substrates or cofactors for epigenetic modifications. These modifications, in turn, lock in a persistent pro-inflammatory gene expression profile. A central focus is the dissection of the ANK2–MAVS–IL-8 axis, a critical link connecting genetic susceptibility (via ANK2 variants) through mitochondrial dysfunction to sustained, IL-8-driven vascular injury. Building on this mechanistic framework, the review explores the translational potential of emerging biomarker candidates (e.g., IL-8, specific methylation marks) and proposes stratified therapeutic strategies that target distinct nodes within this interactive network, including metabolic drivers, epigenetic stabilizers, and inflammatory effectors. Ultimately, this work provides an integrated conceptual and translational roadmap for advancing precision medicine in TAK.

## 1. Introduction

Takayasu arteritis (TAK) is an idiopathic, systemic vasculitis that manifests as chronic granulomatous inflammation of the aorta and its primary branches. The epidemiology of TAK exhibits significant geographic and racial disparities: the estimated annual incidence in Western countries is 1 to 2 per million population, while in Asian populations the prevalence is considerably higher (approximately 40 per million in Japan [1,2], and a period prevalence of 11.72 per million in Shanghai, China). The disease predominantly affects young women, with 90% diagnosed before 40 [3,4], and clinical phenotypes vary among ethnic groups—aortic arch involvement is more frequent in Japanese patients, whereas abdominal aorta involvement is more commonly observed in Indian and Chinese populations [3,5]. Clinical manifestations commonly include acroischemia, diminished pulses, and systemic inflammatory symptoms [6]. Severe cases can lead to arterial stenosis, occlusion, or aneurysm formation that are potentially life-threatening. Pathogenetic research has historically centered on the dysregulated activation of the adaptive immune system, with a predominant focus on the vascular wall infiltration by immune cells—notably T cells and macrophages—and the inflammatory cascade they instigate [7]. Clinical treatment relies on glucocorticoids, immunosuppressants [8], and biologics. However, these therapeutic regimens are frequently hampered by limited efficacy, high relapse rates, and dose-limiting adverse effects, underscoring the absence of reliable predictive biomarkers. Consequently, there is a pressing need to elucidate the underlying pathogenic mechanisms to guide the development of more precise and effective therapies.

Genetic predisposition serves as the foundation for the pathogenesis of TAK. Genome-wide association studies (GWAS) have identified the canonical HLA-B*52:01 allele along with several novel non-HLA susceptibility loci (e.g., VPS8, SVEP1). Functional annotations of these loci suggest a predominant impact on monocyte and B-cell biology [9], with implicated pathways involving transcription factor families such as STAT and RUNX. Genetic risk scores reveal heterogeneity in disease susceptibility across different populations [10]. Furthermore, the genetic architecture of TAK partially overlaps with that of inflammatory bowel disease, suggesting shared mechanisms of immune dysregulation. Notably, specific genetic variants are closely associated with distinct clinical phenotypes. For instance, the Interleukin 12 Subunit Beta (IL-12B) SNP rs6871626 correlates with severe vascular injury, aortic regurgitation, and hypertension [11]; polymorphisms in the tumor necrosis factor alpha (TNF-α)-induced protein 3 (*TNFAIP3*) gene confer an increased risk of TAK [12]; and the co-occurrence of MEFV mutations with HLA-B*52:01 may synergistically drive the disease and influence the therapeutic response to IL-6 blockade [13]. Collectively, these findings underscore the pivotal role of genetic background in shaping the clinical heterogeneity of TAK.

Epigenetic regulation, serving as a molecular bridge linking genetic susceptibility and environmental exposures, is increasingly recognized for its pivotal role in the pathogenesis of TAK. Mechanisms including DNA methylation, histone modifications, and non-coding RNAs convert environmental signals into enduring cellular memory. Research has revealed that Interleukin (IL)-32 and Lymphotoxin-A were genes significantly hypomethylated in CD8 T-cells, which correlates positively with the severity of vascular inflammation. Anti-inflammatory cytokine genes (such as IL-10 and IL1RN) are hypomethylated in γδ T cells, indicating a distinct epigenetic landscape across T-cell subsets [14]. Environmental factors are postulated to synergize with genetic risk by inducing epigenetic reprogramming, ultimately disrupting immune homeostasis and tolerance [9].

**Figure 1:**
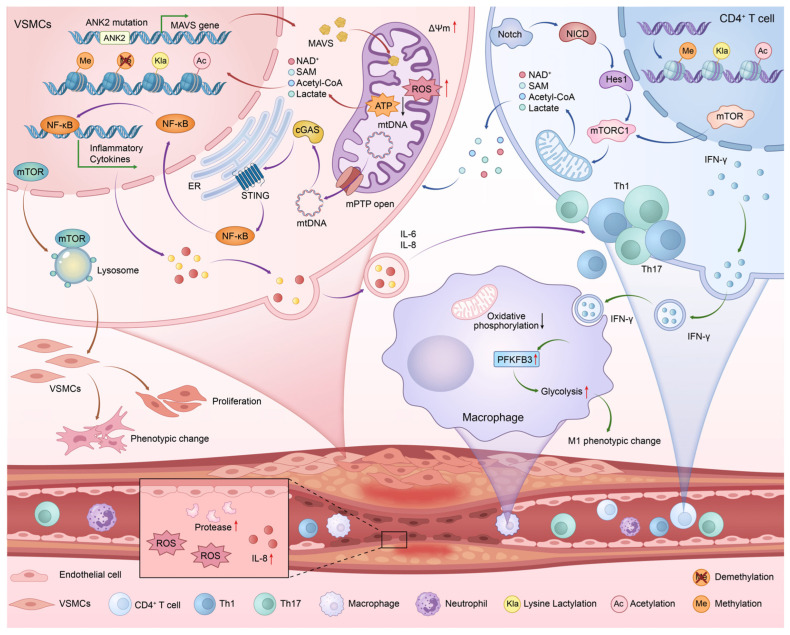
Schematic diagram illustrating the interplay between metabolic reprogramming and epigenetic regulation in the pathogenesis of Takayasu arteritis (TAK). In vascular smooth muscle cells, mutations in the *ANK2* gene lead to mitochondrial stress and hyperpolarization of the mitochondrial membrane potential, inducing the accumulation of reactive oxygen species (ROS) and mitochondrial antiviral-signaling protein (MAVS) oligomerization. Mitochondrial metabolic reprogramming, through metabolites such as acetyl-CoA and lactate, affects epigenetic modifications of DNA and histones, thereby promoting the proliferation and migration of vascular smooth muscle cells. The release of mitochondrial DNA (mtDNA) activates the cGAS-STING signaling pathway, which in turn activates the downstream nuclear factor kappa B (NF-κB) signaling pathway, ultimately leading to increased expression of the pro-inflammatory cytokines IL-6 and IL-8. These pro-inflammatory factors recruit immune cells, such as T cells, neutrophils, and macrophages, causing infiltration of the vessel wall. T cells, regulated by the mTOR signaling pathway, undergo metabolic reprogramming toward glycolysis; their metabolic products are released into the microenvironment and may act on vascular smooth muscle cells to influence their epigenetic modifications. Activated and proliferating T cells release interferon-gamma (IFN-γ), which upregulates 6-phosphofructo-2-kinase/fructose-2,6-bisphosphatase 3 (PFKFB3) activity in macrophages, promoting glycolysis and thereby diminishing oxidative phosphorylation. The persistent crosstalk between vascular smooth muscle cells and immune cells within the vessel ultimately leads to sustained inflammatory cell infiltration, wall thickening, luminal narrowing, and increased vascular permeability.

The interplay between metabolic reprogramming and epigenetic regulation has emerged as a novel paradigm for elucidating the persistence of chronic inflammatory memory. Within the inflammatory microenvironment, metabolites serve as substrates or cofactors for a spectrum of epigenetic modifying enzymes. Reciprocally, epigenetic mechanisms reinforce aberrant metabolic and inflammatory states by modulating the expression of metabolism-related genes. In the context of TAK, vascular wall-resident cells—particularly smooth muscle cells and endothelial cells—manifest substantial metabolic reprogramming driven by ankyrin 2 (*ANK2*) deficiency [15,16], whereas the inflammatory response is amplified by neutrophils, monocytes/macrophages, and T cells. For example, CD4^+^ T cells exhibit metabolic reprogramming, too, evidenced by hyperactivation of the mechanistic target of rapamycin (mTOR) signaling pathway. These metabolic shifts fulfill a dual function: provisioning the energy and biosynthetic precursors requisite for T cell activation and proliferation, and, critically, by yielding specific metabolic intermediates (e.g., acetyl-CoA, α-ketoglutarate, S-adenosylmethionine [SAM]), serving as direct molecular drivers of epigenetic modifications, encompassing DNA methylation, histone acetylation, and lactylation [17,18]. The ensuing epigenetic changes, in turn, consolidate the dysregulated metabolic and inflammatory phenotype, establishing a self-perpetuating “metabolism–epigenetics–inflammation” positive feedback loop (Figure 1). This vicious cycle is postulated to be a core mechanism that underpins the chronic nature and relapse propensity of TAK [19,20,21].

Mitochondrial dysfunction and the ensuing ANK2–MAVS–IL-8 axis represents a critical node within the aforementioned crosstalk. Rare variants in the ankyrin 2 (*ANK2*) gene lead to disorganized mitochondrial cristae structure and membrane hyperpolarization, precipitating a bioenergetic crisis and oxidative stress. This state specifically activates the mitochondrial outer membrane protein MAVS (mitochondrial antiviral-signaling protein). Its oligomerization then triggers the NF-κB signaling cascade, resulting in significant upregulation of IL-6 and IL-8 transcription [15]. As a potent neutrophil chemokine, IL-8 contributes to vascular endothelial injury [22], smooth muscle cell activation, and arterial wall remodeling. An in-depth dissection of this axis not only provides a crucial mechanistic breakthrough for understanding the metabolism–epigenetics interplay in TAK but also lays a solid theoretical foundation for discovering novel biomarkers and developing targeted therapeutic strategies against this interactive network.

This review aims to elucidate the pathogenesis of TAK from the novel perspective of the interplay between metabolism and epigenetics. We will first outline the fundamental roles and interactive networks of metabolic reprogramming and epigenetic regulation in driving TAK-associated vascular inflammation. Subsequently, we will focus on the ANK2–MAVS–IL-8 axis, detailing its mechanism in linking mitochondrial dysfunction to immune-inflammatory responses. Finally, grounded in insights from this axis, we will explore novel combinatorial therapeutic strategies targeting metabolic and epigenetic nodes, as well as the potential for biomarker discovery. We posit that this synthesis will provide a novel conceptual framework and translational directions for the precise diagnosis and treatment of TAK.

## 2. Metabolic Reprogramming Drives Vascular Inflammation in Takayasu Arteritis

### 2.1. Metabolic Reprogramming of Immune Cells

Metabolic reprogramming plays a pivotal role in regulating immune cell function in TAK. The activation and differentiation of immune cells entail a metabolic shift towards metabolic pathways such as glycolysis, oxidative phosphorylation, and fatty acid oxidation [12]. Analogous to the tumor microenvironment, nutrient scarcity and metabolite accumulation within the vascular inflammatory niche can remodel immune cells, inducing further metabolic reprogramming that ultimately impairs their function [13].

Mitochondrial dysfunction acts as a potent inflammatory trigger by increasing the generation of ROS and activating the inflammasome, thereby promoting the release of IL-1β [23]. Furthermore, the release of mitochondrial DNA amplifies immune signaling through the Toll-like receptor (TLR) and cGAS-STING pathways.

### 2.2. Mitochondrial Homeostasis Imbalance and Metabolic Stress

Mitochondria, serving as the cellular powerhouses, are intimately linked to a spectrum of metabolic and inflammatory diseases when altered. Specifically, loss-of-function variants in the *ANK2* gene disrupt mitochondrial cristae architecture and induce membrane hyperpolarization, culminating in bioenergetic failure and diminished ATP production. The resultant mitochondrial membrane hyperpolarization facilitates excessive accumulation of ROS, which exacerbates oxidative stress and inflicts cellular damage. This cascade activates downstream immune signaling pathways and intensifies inflammatory responses. Furthermore, mitochondrial stress can instigate the release of mitochondrial DNA, thereby activating the cGAS-STING pathway and inducing the production of type I interferons and pro-inflammatory cytokines such as IL-6 and IL-8. Within the context of TAK, *ANK2* deficiency promotes MAVS oligomerization, which enhances IL-8 secretion and perpetuates vascular inflammation [15]. Analogous pathophysiological mechanisms are observed in other conditions including metabolic dysfunction-associated steatohepatitis (MASH) and Alzheimer’s disease, underscoring mitochondrial function itself as a promising therapeutic target across metabolic inflammatory disorders [24,25,26,27,28].

### 2.3. Metabolite-Mediated Immunoregulation

Cellular and gut microbial metabolites play pivotal roles in regulating immune cell phenotypes and functions. Metabolites such as lactate and citrate modulate immune cell gene expression and activation states by influencing epigenetic programs and signaling pathways [29,30]. In autoimmune conditions, including rheumatoid arthritis, gut microbiota dysbiosis and consequent metabolite imbalances (e.g., in short-chain fatty acids and tryptophan derivatives) can drive pathogenic pro-inflammatory cytokine secretion [31]. Metabolites exert immunoregulatory effects through receptor binding, transport, and post-translational protein modifications. For instance, the short-chain fatty acid butyrate inhibits histone deacetylase (HDAC), enhancing the differentiation of colonic regulatory T cells [32]. Itaconate, upon uptake by CD8^+^ T cells, suppresses their proliferation, cytokine production, and cytotoxic function by inhibiting key biosynthetic pathways (e.g., for aspartate and serine/glycine), unveiling a novel metabolite-mediated checkpoint mechanism [33]. Furthermore, lactate signals through the GPR81 receptor to modulate immune cell function, and lactate-induced histone lactylation promotes M2 macrophage gene expression, coupling glycolytic flux to epigenetic reprogramming [34,35]. Collectively, these insights establish metabolites as crucial signaling mediators of the immunometabolic microenvironment in TAK.

### 2.4. Metabolic Abnormalities and Endothelial Dysfunction

Metabolic dysregulation significantly compromises endothelial barrier function and increases permeability. In coronary microvascular endothelial cells from diabetic patients, diminished activity of SK potassium channel coincides with an elevated NADH/NAD^+^ ratio and the accumulation of mitochondrial reactive oxygen species. NAD^+^ supplementation restored channel function, suggesting that pyridine nucleotides regulate endothelial ion channel activity through modulation of cellular redox status [36]. Dysregulated lipid metabolism affects endothelial inflammation and barrier integrity by altering the gut–liver axis and inflammatory cytokine expression. For instance, quinolinic acid ameliorates trimethylamine N-oxide (TMAO)-induced lipid metabolism abnormalities and mitigates atherosclerosis [37]. Pigment epithelial-derived factor inhibits Apelin/Apj/VE-cadherin signaling, reducing fatty acid uptake and preventing endothelial junction disruption [38]. In the context of TAK, anti-endothelial cell antibodies target endothelial protein C receptor and scavenger receptor β1, promoting T helper 17 cell (Th17) cell differentiation and endothelial activation [39]. Mast cell-derived histamine and increased indoleamine 2,3-dioxygenase (IDO) enhance vascular permeability and promote fibrotic remodeling [40]. Additionally, exosomes derived from the brown adipose tissue (BAT) of obese individuals downregulate inositol 1,4,5-trisphosphate receptor type 3 (ITPR3), which reduces Ca^2+^ influx and nitric oxide (NO) production, ultimately impairing endothelium-dependent vasodilation [41]. These findings suggest that metabolic abnormalities form a vicious “metabolism–inflammation–vascular injury” cycle via oxidative stress, lipid reprogramming, and immune activation. This mechanistic understanding highlights that targeting metabolic nodes such as SK channels, TMAO, and NADPH oxidase 1 (NOX1) holds promising therapeutic potential.

## 3. Epigenetic Mechanisms Underpin Inflammatory Memory in TAK

### 3.1. DNA Methylation and the Regulation of Inflammatory Gene Expression

DNA methylation, a key epigenetic mechanism, regulates the expression of inflammation-related genes and contributes to the pathogenesis of TAK. In patients with TAK, aberrant DNA methylation in the promoter regions of inflammatory genes significantly alters their transcriptional activity, thereby influencing disease initiation and progression [42]. Typically, promoter hypomethylation of pro-inflammatory genes such as TNF-α and IL-1β leads to their upregulated expression, whereas hypermethylation of anti-inflammatory genes like *IL-10* results in transcriptional silencing. This skewed methylation landscape promotes the activation of pivotal signaling pathways, including NF-κB, and drives the advancement of vascular inflammation [43]. Furthermore, dynamic changes in DNA methylation levels correlate with disease activity. For instance, in smoke-induced inflammation models, global increases in methylation are noted, and differentially methylated genes are enriched in inflammatory pathways. This association highlights the potential of DNA methylation patterns to serve as biomarkers for assessing disease activity in TAK [44].

Aberrant DNA methylation can also serve as a biomarker for disease prognosis. For instance, intermediate DNA demethylation products in peripheral blood mononuclear cells (PBMCs) from metabolic dysfunction-associated steatotic liver disease (MASLD) patients correlate with the risk of liver fibrosis [44], while abnormal DNA methylation patterns in peripheral blood leukocytes of Alzheimer’s disease (AD) patients correlate with systemic inflammatory marker levels [45]. In tuberculosis infection, hypomethylation of the *TNF-α* gene in monocyte-derived dendritic cells (mo-DCs) correlates with bacterial strain drug resistance and demonstrates diagnostic potential [46].

In summary, DNA methylation contributes to TAK pathogenesis through the dysregulated expression of inflammatory genes. The dynamic nature of these epigenetic modifications provides a rich source of potential biomarkers for monitoring disease activity and predicting clinical outcomes. Future research should prioritize the precise mapping of disease-relevant methylation loci and the elucidation of their upstream regulatory networks.

### 3.2. Histone Modifications Regulate Immune Cell Function

Histone modifications, such as acetylation and methylation, precisely regulate inflammatory gene expression by remodeling chromatin structure, thereby exerting a profound influence on immune responses. Within the tumor microenvironment, the expansion of myeloid-derived suppressor cells is intricately linked to specific histone modification states, which guide their differentiation program and reinforce an immunosuppressive phenotype [47]. Histone methylation serves as a critical determinant of immune cells fate, affecting both antitumor immunity and innate immune function [48,49]. In the context of allergic rhinitis, SIN3A regulates CD4^+^ T cell differentiation into Th17 cells while suppressing Tregs, disrupting the T helper 17/regulatory T cell (Th17/Treg) balance [50]. Histone deacetylases (HDACs) negatively regulate immune activation and inflammatory cytokine production by removing acetyl groups to inhibit gene transcription. Consequently, HDAC inhibitors can enhance the efficacy of tumor immunotherapy [51]. Newly identified histone acylation modifications such as lactylation, crotonylation, and β-hydroxybutyrylation link metabolism to epigenetic regulation of immune signaling and immune evasion [52,53]. Therefore, therapeutic targeting of HDACs and novel histone acylation modifications presents innovative strategies for modulating inflammation and advancing immunotherapeutic approaches.

### 3.3. Epigenetic Regulation Mediated by Non-Coding RNAs

Non-coding RNAs (ncRNAs), encompassing microRNAs (miRNAs) and long non-coding RNAs (lncRNAs), modulate immune cell activity by regulating inflammatory signaling molecules, thereby contributing to the pathophysiology of TAK. Specifically, miR-146a/b is dysregulated in the vascular tissues of TAK patients, where it promotes vascular inflammation by targeting intercellular adhesion molecule-1 (ICAM-1). Its expression levels correlate with both disease activity and prognosis, demonstrating potential as a clinical biomarker [54,55,56].

lncRNAs modulate chromatin structure by recruiting histone modification complexes, which subsequently affects endothelial and smooth muscle cell function to promote vascular inflammation and fibrosis [57,58]. Some lncRNAs act as competitive endogenous RNAs, sequestering miRNAs to indirectly regulate target gene expression [59]. The expression profiles of numerous miRNAs and lncRNAs are significantly altered in TAK patients, and these alterations correlate with disease severity and therapeutic response. For instance, *ANK2* gene variants enhance IL-8 secretion via the mitochondrial–immune signaling pathways, thereby promoting vascular inflammation. Abnormalities in non-coding RNAs also influence immune cell activation through epigenetic modifications, driving TAK disease progression and offering novel therapeutic targets [60,61].

### 3.4. The Metabolism–Epigenetics Crosstalk in Solidifying TAK Inflammation

A complex bidirectional feedback loop exists between metabolic reprogramming and epigenetic regulation. On one hand, metabolic intermediates serve as substrates or co-factors for epigenetic modifying enzymes, thereby translating the cellular energy state into heritable gene expression signals [62,63]. Acetyl-CoA is an essential substrate for histone acetyltransferases, directly linking metabolic flux to chromatin architecture, where its abundance dictates the level of histone acetylation [63,64]. Metabolites such as NAD^+^, *α*-ketoglutarate, and S-adenosylmethionine respectively regulate the activities of deacetylases, demethylases, and methyltransferases, affecting DNA and histone modifications [65]. On the other hand, epigenetic mechanisms regulate the expression of metabolic genes, shaping cellular metabolic phenotypes and completing a self-reinforcing regulatory network [62,66]. For example, the histone methyltransferase Enhancer of Zeste Homolog 2 (EZH2) inhibits the transcription of metabolism-related genes via H3K27 trimethylation, driving tumor metabolic reprogramming [21,67]. Metabolic reprogramming of immune cells provides specific metabolites that drive epigenetic remodeling, dictating cellular fate and function [17].

In summary, metabolic dysregulation and epigenetic regulation jointly constitute the core of the pathogenic loop of TAK. The metabolic reprogramming of immune cells profoundly affects the expression of inflammatory genes by regulating key signaling pathways and providing substrates for epigenetic modifications [68]. Epigenetic changes mechanisms may solidify aberrant metabolic and inflammatory states, leading to disease chronicity [69]. Elucidating the key metabolic nodes and epigenetic targets within this loop not only helps reveal the pathogenic nature of TAK but also lays a critical foundation for developing new diagnostic biomarkers and precision therapeutic strategies.

## 4. Role of the ANK2–MAVS–IL-8 Axis in the Pathogenesis of TAK

### 4.1. ANK2 Gene Variants and Mitochondrial Dysfunction

The genetic drivers of TAK, particularly in childhood-onset Takayasu arteritis (c-TAK), remain poorly understood. Recent whole-exome sequencing studies have identified rare *ANK2* gene variants (p.L251I and p.P1306S) in c-TAK patients, closely associated with aggressive renal artery stenosis. The *ANK2* gene encodes ankyrin B, a scaffold protein linking the cytoskeleton to integral membrane proteins. Its dysfunction disrupts mitochondrial cristae architecture and induces abnormal membrane hyperpolarization, inducing ROS production and metabolic stress. This mitochondrial dysfunction activates the MAVS pathway, promoting MAVS oligomerization [15]. This cascade subsequently drives the secretion of pro-inflammatory cytokines IL-6 and IL-8. The released chemokines recruit and activate neutrophils, monocytes and T cells, which amplify and sustain the local inflammation. Thus, rare *ANK2* gene variants in vascular smooth muscle cells (VSMCs) disrupt normal mitochondrial structure and function, triggering a cascade of signals from intracellular metabolic stress to external inflammatory responses. This mechanistic insight uncovers an intrinsic link—a pathogenic continuum—between genetic susceptibility, mitochondrial homeostasis imbalance, and chronic vascular inflammation.

### 4.2. Activation of the MAVS Signaling Pathway Regulates Metabolism and Immunity

MAVS, as the core adaptor molecule in the RIG-I-like receptor (RLR) signaling pathway, requires K63-linked polyubiquitination chain modification for activation. Ube2N and the E3 ligase Riplet/TRIM31 promote non-anchored K63 ubiquitination of MAVS, driving its oligomerization [70]. The deubiquitinating enzyme ubiquitin-specific peptidase 10 (USP10) suppresses MAVS aggregation by removing ubiquitin chains, thereby affecting type I interferon production. Additionally, the mitochondrial-localized protein aggregates promote MAVS phase separation, enhancing its signaling capacity [71]. MAVS links metabolism and immunity by regulating proinflammatory factors such as IL-6 and IL-8. The SARS-CoV-2 non-structural protein nonstructural protein 5 (Nsp5) exacerbates inflammation by augmenting SUMOylation, thereby activating the NF-κB pathway [72]. Phosphorylation also participates in regulation: the p38 MAPK pathway, via the kinase ASK1, phosphorylates MAVS to enhance its interaction with TRAF and amplify the type I interferon response [73]. Conversely, methylation mediated by protein arginine methyltransferase 9 (PRMT9) inhibits MAVS spontaneous activation [74]. These mechanisms delineate the complex regulatory network positioning MAVS as a pivotal hub integrating metabolic and immune signals [75].

### 4.3. IL-8 as a Biomarker in Vascular Inflammation and Renal Artery Stenosis

As a key proinflammatory chemokine, IL-8 plays a pivotal role in renal artery stenosis (RAS). It activates neutrophils and monocytes, promoting their adhesion to the vascular endothelium and subsequent release of ROS and proteolytic enzymes, leading to endothelial cell injury and increased vascular permeability [76,77]. Furthermore, IL-8, secreted by ANK2-deficient VSMCs, induces migration and proliferation of VSMCs themselves and promotes the infiltration of neutrophils/macrophages into the vascular wall [78], accelerating arterial wall remodeling and luminal narrowing (Figure 1). Clinical data show that serum IL-8 levels in TAK patients positively correlate with renal artery stenosis severity, and ANK2 variant carriers exhibit higher IL-8 secretion, supporting the notion that the ANK2–MAVS–IL-8 axis drives vascular inflammation [15]. In preclinical models, IL-8 promotes the expression of vascular cell adhesion molecules, enhances leukocyte infiltration and local inflammation, and activates NF-κB and MAPK signaling pathways, triggering an inflammatory cascade [79]. IL-8 shows potential as a biomarker in coronary artery lesions and in predicting responses to immunotherapy [80]; its specificity requires further refinement. Future studies should validate its application value in the stratified diagnosis and management of RAS, as well as in guiding targeted therapeutic interventions [81,82].

## 5. Combined Therapeutic Strategies Targeting Metabolism and Epigenetic Regulation and the Application of Biomarkers in TAK

### 5.1. Metabolism-Targeted Therapeutic Strategies

In recent years, metabolic reprogramming has emerged as a critical factor in autoimmune diseases, particularly in chronic large-vessel inflammatory disorders such as TAK. Studies have demonstrated that modulation of mitochondrial function and metabolic pathways can effectively attenuate immune-inflammatory responses in TAK patients. Pathological analysis of TAK vasculature reveals increased infiltration of M1 macrophages, a phenomenon that correlates with elevated expression of interferon-γ (IFN-γ). IFN-γ upregulates the glycolytic rate-limiting enzyme PFKFB3 via the JAK2/STAT1 signaling pathway, thereby promoting M1 macrophage polarization [83]. Consequently, PFKFB3 inhibitors, which can significantly suppress IFN-γ-induced glycolysis and M1 polarization, may represent a promising novel therapeutic strategy for TAK.

Immunosenescent T cells secrete high levels of osteopontin in conditions like obesity and aging, contributing to metabolic dysfunction and chronic inflammation. Therefore, mitochondrial protective agents aimed at improving mitochondrial function may help attenuate vascular inflammation in TAK patients. Additionally, glycolysis inhibitors reduce inflammatory cytokine production and curb M1 macrophage polarization by targeting glycolytic pathways [83].

Potential therapeutic agents include the JAK inhibitor tofacitinib and the IL-6-targeted monoclonal antibody tocilizumab, both of which have demonstrated clinical efficacy in TAK. Future studies should explore the intricate interplay between metabolic dysregulation and epigenetic control in TAK to guide the development of more effective combination therapies.

### 5.2. Epigenetic Therapeutic Strategies

Histone deacetylase (HDAC) inhibitors and DNA methyltransferase (DNMT) inhibitors, regulate expression of inflammatory mediators by remodeling the cellular epigenetic landscape. Studies have shown that HDAC inhibitors can downregulate the expression of pro-inflammatory mediators like hypoxia-inducible factors 1-alpha (HIF-1α) and IL-1β while upregulating anti-apoptotic genes, including BCL2-like 13 (*BCL2L13*) and *CASPASE-9* [84]. DNMT inhibitors function by reversing aberrant DNA methylation, thereby restoring the expression of genes critical for processes such as bone metabolism [85]. These epigenetic drugs often exhibit pleiotropic effects and possess epigenetic reprogramming properties. For example, trichostatin A has been shown to synergize with anti- vascular endothelial growth factor (VEGF) therapies to regulate oxidative stress-related genes [84]. However, challenges remain regarding dose optimization and specificity.

Non-coding RNAs have gained increasing attention as key mediators of epigenetic regulation and potential therapeutic targets. Long non-coding RNAs can regulate drug-metabolizing enzymes and transporters by influencing histone modifications and DNA methylation patterns [86]. MicroRNAs (miRNAs) exert post-transcriptional control over enzyme systems such as Cytochrome P450 (CYP450), playing a crucial role in mechanisms like tumor drug resistance [87]. ncRNA-based therapeutic strategies include the use of miRNA mimics or inhibitors, small molecules or antisense oligonucleotides targeting specific lncRNAs, and engineered delivery systems. Despite this promise, the path to clinical application is obstructed by considerable hurdles, including suboptimal delivery efficiency, inadequate tissue specificity, and unresolved long-term safety concerns. These challenges necessitate continued efforts in the optimization of design and thorough evaluation.

### 5.3. Combined Metabolic and Epigenetic Therapeutic Strategies

Metabolic and epigenetic regulation form a complex interactive network in TAK that establishes the basis for combination therapy. Metabolic intermediates such as acetyl-CoA can directly modulate the activity of epigenetic enzymes [88], whereas epigenetic modifications can in turn regulate metabolic enzyme expression [89]. This reciprocal regulatory mechanism provides a robust foundation for multi-targeted therapeutic interventions. Current combined strategies mainly encompass: co-administration of metabolic modulators and epigenetic drugs; dual-function small molecules (e.g., melatonin); and nanotechnology-based co-delivery systems. These approaches warrant exploration in large-vessel vasculitis, particularly for targeting persistent fibroblast activation within the vascular wall. Achieving precision and individualized treatment will necessitate the development of reliable biomarkers and the optimization of drug administration timing [90]. Future research should integrate single-cell multi-omics technologies to characterize metabolic–epigenetic heterogeneity in TAK, thereby enabling the design of dynamic therapeutic strategies tailored to different disease stages (active versus remission phases).

### 5.4. Development and Clinical Application of Biomarkers

The development of metabolite and epigenetic biomarkers is of paramount importance for the clinical management of TAK. IL-8, a pivotal chemokine at the metabolism-related inflammatory factor, shows markedly elevated serum levels and correlates positively with disease activity scores, demonstrating its considerable potential as a clinical biomarker. Compared with IL-6 and TNF-α, IL-8 exhibits superior specificity for vascular lesions [91].

Epigenetic biomarkers also demonstrate value in early diagnosis and therapeutic monitoring. Peripheral blood lncRNAs in TAK patients—including THRIL, HIF1A-AS1, and HOTAKIR—are significantly downregulated. This dysregulation confers high diagnostic specificity (AUC > 0.78) and is implicated in pathogenesis likely through the modulation of immune-inflammatory pathways [92]. DNA methylation markers such as *CYP2E1* and *ZFPM2* correlate with both disease activity and vascular damage, highlighting the critical role of epigenetic modifications in TAK pathology [93].

Combined biomarker strategies further improve diagnostic and monitoring performance. For example, combined detection of serum complement C3 and C-reactive protein increases sensitivity to 85.1% and specificity to 94.1% for disease activity assessment [94]. Moreover, integration of positron emission tomography–computed tomography (PET-CT) vascular activity scores (PETVAS) with novel circulating markers, such as pentraxin 3 (PTX3), improves the prediction of new vascular lesions and the risk of adverse events in TAK patients [95]. This multimodal biomarker strategy not only mitigates the limitations inherent to single-analyte approaches but also opens new avenues for precision medicine in TAK.

In conclusion, the metabolic-inflammatory and epigenetic markers provide important tools for the early diagnosis, assessment of disease activity, and monitoring of treatment response of TAK. Different targeting strategies should be developed for different pathogenic tiers (Table 1). Future research should further validate the clinical applicability of these markers and explore their potential in individualized treatment.

## 6. Research Methods and Technological Advances in Metabolic and Epigenetic Regulation

### 6.1. Multi-Omics Integration Technologies and Applications

High-throughput sequencing technologies have driven the development of single-cell multi-omics approaches—including RNA sequencing, proteomics, metabolomics, and chromatin accessibility sequencing—providing multilayered insights into the pathogenesis of large-vessel vasculitis. Cross-omics data integration methods, such as single-cell optimal transport (SCOT) algorithm and the cross-attention mechanism-based deep learning architectures CrossAttOmics framework, enable unsupervised alignment and fusion of heterogeneous omics datasets, thereby enhancing the accuracy of disease subtyping and outcome prediction [96,97]. The Windows Scanning Multiomics (WSM) technology facilitates the simultaneous extraction of metabolites and proteins. When coupled with ultra-performance liquid chromatography-mass spectrometry (UPLC-MS) analysis, it constructs more precise metabolite–protein association networks while mitigating false-positive correlations inherent in conventional methods [98]. Additionally, approaches based on Boolean networks (e.g., mBNITA), coupled with software packages from the Bioconductor project, facilitate the standardized integration and analysis of multi-omics data, thereby significantly streamlining analytical workflows for both multi-omics and pan-cancer studies [99,100]. Multi-omics studies in COVID-19 have also demonstrated the potential of integrating transcriptomics, proteomics, and lipidomics to elucidate complex disease mechanisms [101].

Artificial intelligence, particularly deep learning, is further driving innovative integration of multi-omics data. The SUPREME framework utilizes graph convolutional neural networks to integrate multi-omics features, achieving precise prediction of breast cancer subtypes [102]. Meanwhile, models based on t-distributed stochastic neighbor embedding (t-SNE) and uniform manifold approximation and projection (UMAP) support nonlinear dimensionality reduction and cross-omics projection of large-scale mass spectrometry imaging and chromosome conformation capture datasets [103]. The BaySyn framework utilizes hierarchical Bayesian models to identify functionally relevant driver genes, uncovering lineage-specific biomarkers in gynecological cancers [104]. Emerging tools such as scPairing and spatial multi-omics technologies like INLAomics are expected to enhance multimodal data integration and spatiotemporal analysis [105,106]. Dynamic interactions between metabolism and epigenetics, such as lactylation-mediated histone modification regulated via the GPR81/GPR4 signaling pathway, highlight coupling mechanisms between metabolic flux and chromatin remodeling, providing a new conceptual lens for investigating immunometabolic dysregulation in TAK [107].

### 6.2. Single-Cell Technologies Reveal Cellular Heterogeneity and Metabolic–Epigenetic Interactions

Single-cell RNA sequencing and single-cell chromatin accessibility sequencing technologies provide high-resolution tools for dissecting immune cell subpopulations in TAK. scRNA-seq has elucidated the transcriptional heterogeneity of T cells, B cells, and macrophage subsets infiltrating the vascular wall [108], while scATAC-seq complements these findings by identifying chromatin accessibility landscapes and identifying immune cell-specific regulatory regions [109]. Multi-omics integration strategies further deepen our understanding of epigenetic regulatory networks in immune cells.

Cell-type-specific disparities in metabolic and epigenetic regulation constitute pivotal drivers of TAK pathogenesis. Single-cell multi-omics studies demonstrate that distinct metabolic states, such as enhanced glycolysis, are closely associated with histone lactylation, which regulates pro-inflammatory gene expression [110,111]. Single-cell metabolomics tools such as single-cell flux estimation analysis (scFEA) enable quantification of metabolic flux at the cellular level, thereby uncovering metabolic dependencies of pro-inflammatory macrophages [112]. These findings suggest that targeting metabolic–epigenetic crosstalk within specific cellular subpopulations may represent a novel therapeutic strategy for TAK.

### 6.3. Applications of Bioinformatics and Systems Biology Approaches

Bioinformatics and systems biology approaches integrate multi-omics data with machine learning to construct metabolic–epigenetic interaction networks, revealing key molecular circuits in TAK. Transcriptomic analyses have identified significant elevation of chemokines such as CCL2, CCL20, CXCL8, and CXCL10 in plasma from TAK patients. These levels show strong correlations with disease activity, and their combined measurement demonstrates high diagnostic value [18]. Network analysis has demonstrated that mutations in the *NSD1* gene influence tumor metabolic phenotypes by regulation of the TGFB2/PPARGC1A signaling axis, highlighting the impact of metabolic heterogeneity on therapeutic outcomes [113]. The *DLST* gene promotes tumor progression by regulating histone demethylase activity, suggesting that pivotal nodes at the metabolism–epigenetics interface represent promising therapeutic targets [114].

In the realm of therapeutic target prediction, bioinformatics tools have been instrumental in uncovering epigenetic modifications associated with the PI3K/Akt pathway-related genes and circular RNAs (circRNAs), revealing their respective roles in tumor progression [115,116]. Within TAK research, screening of lactylation-related genes identified the RBM25-ACLY axis as a driver of metabolic–epigenetic reprogramming via histone lactylation [117]. Furthermore, age-associated DNA methylation changes influence gene expression, suggesting that epigenetic aging contributes to TAK pathogenesis [118]. Future integration of single-cell multi-omics and spatial transcriptomics technologies will enable detailed analysis of how metabolites such as lactate drive inflammatory responses through histone modifications or DNA methylation, providing theoretical foundations for precision therapeutic strategies.

### 6.4. Functional Validation Using Cell and Animal Models

While the studies reviewed above establish strong associations between the ANK2–MAVS–IL-8 axis and TAK pathogenesis, causal evidence is essential to validate this pathway as a therapeutic target. However, the field currently lacks ideal animal models that fully recapitulate TAK pathogenesis [119]. Recent functional studies using ANK2-knockdown models have provided direct mechanistic evidence: ANK2 deficiency induces mitochondrial cristae disorganization and membrane hyperpolarization, leading to MAVS oligomerization and enhanced IL-8 secretion [15]. Existing models include human artery xenografts in immunodeficient mice, which maintain arteritis and enable therapeutic testing [120]. Animal models have also been developed by injecting Toll-like receptor (TLR) ligands into the perivascular adipose tissue (PVAT) surrounding selected segments of the aorta [119] in genetically modified mice, such as mice deficient in interferon regulatory factor (IRF)-4 binding protein (IBP) [121] and interleukin-1 receptor antagonist-deficient (Il1rn−/−) mice [122]. These mice develop inflammation in the aorta.

## 7. Future Research Directions and Challenges

Although metabolic–epigenetic regulatory networks offer a novel paradigm for understanding TAK, translating this knowledge into clinical benefits remains a core challenge. Decoding spatiotemporal dynamics and cellular heterogeneity presents the primary bottleneck. Current research, which predominantly relies on analyses of circulating cells or tissue homogenates, fails to capture the real-time metabolic–epigenetic crosstalk within the local vascular wall microenvironment, particularly at the active inflammatory front. Therefore, the development and application of spatial multi-omics technologies—such as the integration of spatial metabolomics with ATAC-seq—coupled with advanced in vivo imaging techniques (e.g., reporter gene systems for tracing specific metabolites) are imperative. These approaches are key to in situ mapping of the interactive states among distinct cellular subpopulations (e.g., resident fibroblasts versus infiltrating lymphocytes) within diseased vasculature, thereby revealing the intricate “geography” of the disease.

Second, establishing predictive models and functional validation systems is urgently needed. Potential targets identified through multi-omics data screening—such as specific histone lactylases—require rigorous causal validation. This necessitates the use of sophisticated models that faithfully recapitulate the human disease milieu, such as patient-derived vascular organoids or humanized mouse models of chronic vascular wall inflammation. To further validate the ANK2–MAVS–IL-8 axis, future studies should consider generating vascular-specific ANK2 knockout mouse models to determine whether ANK2 deficiency alone can trigger large vessel inflammation and testing MAVS inhibitors or IL-8-neutralizing antibodies in existing vasculitis models.

Concurrently, developing AI-driven dynamic predictive models that integrate longitudinal metabolomics, epigenetic markers, and imaging features is crucial for discerning the transition from active to chronic phases and predicting treatment resistance.

Ultimately, the overarching goal is to achieve truly individualized and dynamic therapeutic intervention. This requires us to transcend “static classification” and explore sequential therapeutic strategies tailored to distinct disease phases—such as the acute inflammatory stage versus chronic fibrosis. For instance, early interventions could target metabolic reprogramming to “erase” inflammatory memory, while later stages could focus on the epigenetically maintained fibrotic programs.

Furthermore, exploring how modulation of the gut microbiota and its metabolome can indirectly reshape the host’s metabolic–epigenetic axis may unveil novel, non-immunosuppressive adjunctive avenues for TAK management. Beyond epigenetic modifications, protein glycosylation constitutes an additional layer of post-translational regulatory mechanisms that integrate metabolic signals with immune function. Studies have shown that aberrant IgG glycosylation exists in patients with certain autoimmune diseases and vasculitis [123,124], including TAK [125], and is associated with disease activity [126]. Metabolic reprogramming may drive these alterations, positioning glycosylation as a potential complementary biomarker and novel therapeutic target.

## 8. Discussion and Conclusions

The reciprocal interplay between metabolic reprogramming and epigenetic regulation constitutes a core mechanism underpinning the chronic inflammatory state in TAK [19,20,127]. Herein, we articulate that in TAK, metabolic reprogramming of VSMCs and endothelial cells serves a dual function: it furnishes the bioenergetic and biosynthetic precursors required for cell activation while simultaneously reshaping the intracellular metabolome. These metabolites, acting as substrates or co-factors, directly modulate the activity of epigenetic enzymes, thereby shaping the long-term transcriptional landscape of inflammation-related genes and establishing a persistent inflammatory phenotype [128]. This metabolic–epigenetic crosstalk forms a self-reinforcing “inflammatory memory,” enabling sustained vascular inflammation and fibroblast activation even after inflammatory triggers subside [21,129]. Recent elucidation of the ANK2–MAVS–IL-8 axis provides crucial insights into how mitochondrial metabolic stress arising from genetic susceptibility is translated into sustained immune-inflammatory responses via specific signaling pathways in TAK.

Rare variants in the *ANK2* gene in vascular smooth muscle cells (VSMCs) lead to mitochondrial cristae disorganization and dysfunction, inducing abnormal mitochondrial membrane potential, reactive oxygen species accumulation, and a bioenergetic crisis [15,25]. This state of mitochondrial stress specifically activates the mitochondrial-anchored adaptor protein MAVS. Upon oligomerization, MAVS triggers a downstream signaling cascade, including the NF-κB pathway, driving excessive secretion of key proinflammatory cytokines such as IL-6 and IL-8 from the affected vascular cells [15,76]. Among these, IL-8, a potent neutrophil chemoattractant and activator, is central to vascular endothelial injury, smooth muscle cell activation, and arterial wall remodeling [81,130]. Thus, the ANK2–MAVS–IL-8 axis represents a critical link connecting genetic susceptibility, mitochondrial dysfunction, and chronic vascular inflammation.

**Figure 2:**
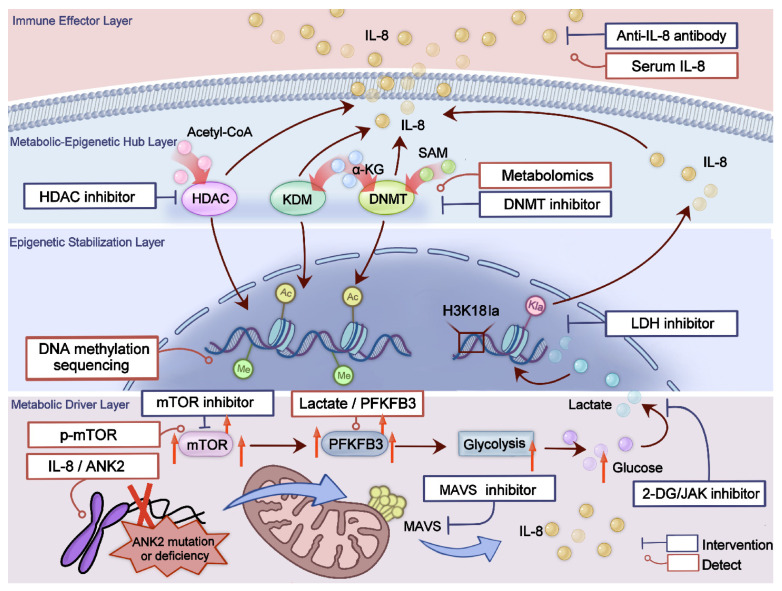
From molecular mechanisms to precision interventions: a conceptual framework for mechanistic risk stratification. This schematic integrates four pathogenic layers: metabolic driver (ANK2–MAVS, mTOR, PFKFB3 axes), epigenetic stabilization (histone lactylation, DNA methylation), metabolic–epigenetic hub (metabolite-enzyme coupling), and immune-inflammatory effector (IL-8). For each, key molecules, measured parameters, and potential targeted interventions are aligned.

From a clinical translation perspective, elucidating this axis holds multiple implications. First, it broadens the traditional perspective of immune cell infiltration, partially tracing the root cause of the disease to organelle dysfunction and metabolic imbalance. Second, IL-8, as a key output molecule of this pathway, demonstrates significant potential as a specific biomarker for disease activity and vascular injury. Clinical studies confirm its serum levels correlate positively with the severity of renal artery stenosis and are elevated in ANK2 variants, facilitating more precise risk stratification and treatment monitoring [85]. Third, the development of multiple targets within this axis offers novel therapeutic strategies for TAK management [80]. To further illustrate the translational potential of this metabolic–epigenetic crosstalk framework, we propose a molecular and cellular mechanistic approach to patient stratification based on the distinct pathogenic layers discussed above (Figure 2). Such strategies may exhibit synergistic effects when combined with traditional immunosuppressants or JAK inhibitors, offering particular benefit for patients with inadequate response or high recurrence risk to existing therapies [131].

Notably, the metabolic–epigenetic interaction pattern is not exclusive to TAK. A similar co-occurrence of enhanced glycolysis, mitochondrial dysfunction, and aberrant epigenetic modifications like histone lactylation has been observed in other autoimmune diseases, such as rheumatoid arthritis and giant cell arteritis [57,113,132]. This suggests that therapeutic strategies targeting shared nodes—such as the key metabolic enzyme PFKFB3 and epigenetic regulators HDACs—have broad-spectrum potential across related diseases, providing theoretical support for drug repurposing [56].

However, translating this cutting-edge knowledge into clinical practice remains challenging. The TAK patient population exhibits significant heterogeneity, with immunometabolic and epigenetic profiles varying according to disease stage, affected vascular bed, and individual genetic background [114,133]. Future research must leverage advanced technologies such as single-cell multi-omics and spatial transcriptomics to dissect the specific metabolic–epigenetic dialogs between distinct cellular subpopulations (e.g., infiltrating immune cells and fibroblasts) within the vascular wall microenvironment across both spatial and temporal dimensions [102,103]. Concurrently, large-scale longitudinal cohort studies are essential to validate the predictive value of biomarkers related to the ANK2–MAVS–IL-8 axis for disease relapse, treatment response, and vascular progression. Furthermore, developing integrated predictive models that combine metabolomic signatures (e.g., lactate, acetyl-CoA) with epigenetic markers (e.g., specific lncRNAs or DNA methylation sites) represents a promising direction [18].

In conclusion, the therapeutic paradigm for TAK is poised to evolve from simple immunosuppression toward a multidimensional strategy that integrates metabolic modulation, epigenetic intervention, and precision immune targeting. By deciphering and therapeutically targeting core interaction networks such as the ANK2–MAVS–IL-8 axis, we can envisage disrupting the vicious cycle of chronicity and recurrence in TAK, ultimately improving long-term patient outcomes.

## Figures and Tables

**Table 1:** Stratified targeting strategies for the metabolic–epigenetic–immune network in Takayasu arteritis.

Pathogenic Layer	Functional Module	Key Molecules	Mechanisms	Pathological Consequences	Potential Targeting Strategies
Metabolic Driver Layer	Mitochondrial Stress	*ANK2*, MAVS	Inflammatory Signaling Activation	IL-8 elevation, vascular stenosis	MAVS inhibitors, Mitochondrial protective agents
	T-cell Metabolism	mTOR	Th1/Th17 Differentiation	Immune Infiltration	mTOR inhibitors
	Glycolytic Reprogramming	PFKFB3	M1 Macrophage Polarization	Inflammatory amplification	Glycolysis inhibitors
Epigenetic Stabilization Layer	Histone Modifications	H3K18la/H4K12la	Inflammatory Gene Transcription	Chronic inflammation	LDH inhibitors, Lactylation-targeting strategies
	DNA Methylation	Pro-inflammatory Genes	NF-κB-driven Inflammation	Immune imbalance	DNMT inhibitors
	ncRNAs	miR-146a/b Axis	ICAM-1 Modulation	Endothelial injury	miRNAmimics/inhibitors
Metabolic–Epigenetic Hub Layer	Metabolite-Chromatin interaction	Acetyl-CoA/α-KG/SAM	Cofactors	transcriptional reprogramming	Metabolite supplementation or antagonism
	Deacetylation Sensing System	Sirtuins/HDACs	Metabolic regulation of chromatin	Immune imbalance	HDAC inhibitors
Immune-Inflammatory Effector Layer	Cytokine Amplification Loop	MAVS–IL-8 Axis	Neutrophil Recruitment	Vascular injury and stenosis	Anti-IL-8 monoclonal antibodies, JAK inhibitors
	T-cell Inflammatory Circuit	Notch1–mTORC1 Axis	Locked Inflammatory Phenotype	Chronic inflammation	Notch1 inhibitors

## Data Availability

No new data were created or analyzed in this study. Data sharing is not applicable to this article.

## Abbreviations

Listed below are the abbreviations that appear in this manuscript:

α-KG	alpha-ketoglutarate
ANK2	ankyrin 2
BCL2L13	BCL2-like 13
cGAS	cyclic GMP–AMP synthase
c-TAK	childhood-onset Takayasu arteritis
CYP450	Cytochrome P450
DNMT	DNA methyltransferase
EZH2	enhancer of zeste homolog 2
HDAC	histone deacetylase
HIF-1α	hypoxia-inducible factor 1-alpha
H3K18la	Histone H3 lysine 18 lactylation
IDO	indoleamine 2,3-dioxygenase
IFN-γ	interferon-gamma
IL-1β	interleukin-1 beta
IL-6	interleukin-6
IL-8	interleukin-8
IL-12B	Interleukin 12 Subunit Beta
LDH	lactate dehydrogenase
MAVS	mitochondrial antiviral-signaling protein
mTOR	mechanistic target of rapamycin
mTORC1	mechanistic target of rapamycin complex 1
NF-κB	nuclear factor kappa B
Notch1	neurogenic locus notch homolog protein 1
NOX1	NADPH oxidase 1
Nsp5	nonstructural protein 5
PFKFB3	6-phosphofructo-2-kinase/fructose-2,6-bisphosphatase 3
PRMT9	protein arginine methyltransferase 9
ROS	reactive oxygen species
SAM	S-adenosylmethionine
scFEA	single-cell flux estimation analysis
STING	stimulator of interferon genes
TAK	Takayasu arteritis
Th1	T helper 1 cell
Th17	T helper 17 cell
TLR	Toll-like receptor
TMAO	trimethylamine N-oxide
TNF-α	tumor necrosis factor alpha
TNFAIP3	TNF-α-induced protein 3
Treg	regulatory T cell
USP10	ubiquitin-specific peptidase 10
VEGF	vascular endothelial growth factor
ZFPM2	zinc finger protein, FOG family member 2
